# Current perspectives on the regulatory mechanisms of sucrose accumulation in sugarcane

**DOI:** 10.1016/j.heliyon.2024.e27277

**Published:** 2024-02-28

**Authors:** Faisal Mehdi, Saddia Galani, Kamal Priyananda Wickramasinghe, Peifang Zhao, Xin Lu, Xiuqin Lin, Chaohua Xu, Hongbo Liu, Xujuan Li, Xinlong Liu

**Affiliations:** a Sugarcane Research Institute, Yunnan Academy of Agricultural Sciences/Yunnan Key Laboratory of Sugarcane Genetic Improvement, Kaiyuan, Yunnan 661699, China; b National Key Laboratory for Tropical Crop Breeding, Key Laboratory of Biology and Genetic Resources of Tropical Crops (Ministry of Agriculture and Rural Affairs), Institute of Tropical Bioscience and Biotechnology, Sanya Research Institute, Chinese Academy of Tropical Agricultural Sciences, Haikou 571101, China; c Dr.A. Q. Khan Institute of Biotechnology and Genetic Engineering, University of Karachi, Karachi Pakistan; d Sugarcane Research Institute, Uda Walawa, 70190, Sri Lanka

**Keywords:** Sucrose metabolizing enzymes, Source-sink communication, Post-transcriptional factors, Sucrose accumulation, Sugarcane genetic engineering

## Abstract

Sugars transported from leaves (source) to stems (sink) energize cell growth, elongation, and maintenance. which are regulated by a variety of genes. This review reflects progress and prospects in the regulatory mechanism for maximum sucrose accumulation, including the role of sucrose metabolizing enzymes, sugar transporters and the elucidation of post-transcriptional control of sucrose-induced regulation of translation (SIRT) in the accumulation of sucrose. The current review suggests that SIRT is emerging as a significant mechanism controlling *Scbzip44* activities in response to endogenous sugar signals (via the negative feedback mechanism). Sucrose-controlled upstream open reading frame (SC-uORF) exists at the 5′ leader region of *Scbzip44*'s main ORF, which inhibits sucrose accumulation through post-transcriptional regulatory mechanisms. Sucrose transporters (*SWEET1a/4a/4b/13c, TST, SUT1, SUT4 and SUT5*) are crucial for sucrose translocation from source to sink. Particularly, *SWEET13c* was found to be a major contributor to the efflux in the transportation of stems. Tonoplast sugar transporters (TSTs), which import sucrose into the vacuole, suggest their tissue-specific role from source to sink. Sucrose cleavage has generally been linked with invertase isozymes, whereas sucrose synthase (SuSy)-catalyzed metabolism has been associated with biosynthetic processes such as UDP-Glc, cellulose, hemicellulose and other polymers. However, other two key sucrose-metabolizing enzymes, such as sucrose-6-phosphate phosphohydrolase (S6PP) and sucrose phosphate synthase (SPS) isoforms, have been linked with sucrose biosynthesis. These findings suggest that manipulation of genes, such as overexpression of SPS genes and sucrose transporter genes, silencing of the SC-uORF of *Scbzip44* (removing the 5′ leader region of the main ORF that is called SIRT-Insensitive) and downregulation of the invertase genes, may lead to maximum sucrose accumulation. This review provides an overview of sugarcane sucrose-regulating systems and baseline information for the development of cultivars with higher sucrose accumulation.

## 1 Introduction

Sugarcane (*Saccharum* spp.) is a significant sugar and energy crop. Approximately 111 countries throughout the world grow sugarcane, which 80% of the sugar and 40% of the ethanol consumed globally [1]. World sugar output is expected to be 179.6 million metric tons in the 2022–2023 year and consumption climbed by 0.6% from the previous year [2]. The demand for sugar in the world increases year by year, but the area of sugarcane planting is limited, so increasing the sugar content of sugarcane is an effective measure to solve this problem [3]. Photosynthesis is the main source of fixed Carbon for all life on Earth and is performed by plants, algae and cyanobacteria. The primary type of sugar that plants synthesize, transport and store is sucrose, one of the final products of photosynthesis [4]. Sucrose is an important signaling molecule affecting plant growth and development, including physiological processes, photosynthesis and Carbon partition [5]. Sugars transported from source (leaves) to sink (stem) provide energy for cell growth, elongation and maintenance and the remaining sugars are stored in vacuoles in the form of sucrose. Sugarcane plants have developed complex systems to detect, process and store sucrose and harmonize their accessibility with the various developmental processes for consumption during crop development. Sucrose concentration in sugarcane stems depend on photosynthesis, metabolism and sink strength or sink limits. The photosynthetic activity decreases as large concentrations of sucrose build up in the stems. Sucrose-metabolizing enzymes regulate the sucrose accumulation in sugarcane. Many plant species have been examined for the genes involved in sucrose accumulation, including source-sink signal transduction molecules, sucrose carriers, zip family genes and sucrose metabolizing enzymes [6]. Therefore, attention has been drawn to the study of the sugarcane's regulation mechanisms for maximum sucrose accumulation. Despite the fact that sugarcane crops are good for sugar production, it is still unclear what underlying sucrose regulation mechanism controls how much sucrose may accumulate in sugarcane stems. So, it is necessary to elucidate the sucrose regulatory mechanisms, which will be useful to improve sugarcane yield along with the development of cultivars with higher sucrose content. The current review primarily concentrates on the most recent developments in sugar regulating systems for maximum sucrose content in sugarcane crops from a variety of perspectives, including the following: (i) sucrose biosynthesis; (ii) source-sink regulation of sucrose accumulation; (iii) post-transcriptional control of sucrose; (iv) role of sugar carriers or transporters; and (v) role of sucrose metabolizing enzymes. This review will help to decipher the regulation of sucrose synthesis, transportation and storage, which are essential for more efficient sucrose accumulation in sugarcane plants.

## 2 Sucrose regulatory mechanisms in sugarcane

### 2.1 Sucrose biosynthesis

**Fig. 1:**
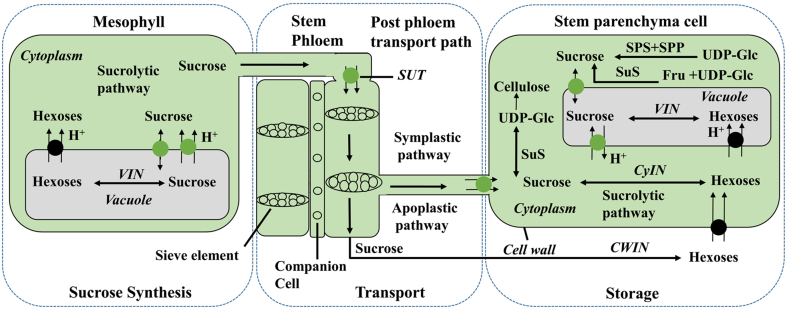
A potential pathway for sucrose transportation in a sugarcane. Sucrose synthesizes in sugarcane leaf mesophyll cells and is temporarily stored in leaf vacuoles. Further, the sucrose is transported by the transporter gene through either the symplastic pathway or the apoplastic pathway. During transportation, sucrose is hydrolyzed into hexose (fructose and glucose) at different locations by invertases like the cell wall, cytoplasmic, and vacuolar invertases. In the cytoplasm, sucrose is resynthesized by SPS and SPP. Finally stored in vacuoles. **Abbreviations:** Cytoplasmic invertase (CyIN), vacuolar invertase (VIN), cell wall invertase (CWIN), sucrose synthase (SuS), sucrose phosphate synthase (SPS), sucrose phosphate phosphatase (SPP), sucrose transporters (SUTs), Fructose (Fru), uridine diphosphate glucose (UDP-Glc). **Note:** The green circle indicates sucrose transporters and the black circle indicates hexose transporters, while the arrow indicates sucrose flow from source to sink.

Sugarcane, a C4 plant, converts light energy into chemical energy through photosynthesis. The partitioning of carbon (C) is crucial for allocating this energy. The primary locations for photosynthesis and carbon assimilation are bundle sheath cells and chloroplasts. The C4 cycle operates in mesophyll cells, while the C3 cycle functions in bundle sheath cells, known as photosynthetic carbon assimilation (PCA) [7]. During photosynthesis, sugarcane plants produce triphosphate in the chloroplast. This triphosphate is moved to the cytoplasm matrix (cytosol), where sucrose and hexose phosphate are produced and then transported to the stems. The condensation of two triphosphates forms fructose 1, 6-biphosphate, which is hydrolyzed to yield fructose-6-phosphate. Sucrose-6-phosphate synthase then catalyzes the reaction of fructose 6-phosphate with UDP-Glc to form sucrose -6-phosphate [8]. Mostly, the triose phosphate produced by the CO_2_ fixation process is transformed into sucrose. Sucrose can be translocated from source to sink through the apoplastic and symplastic pathways [9]. Sucrose can be cleaved in the apoplast by acid invertase (cell wall invertase) to yield hexose. This hexose is then transported via hexose transporters [10]. During the transportation of the apoplastic pathway, sucrose is hydrolyzed by cell wall invertase into hexose and hexose enters the cytoplasm. On the other hand, sucrose enters the cytoplasm (via sucrose transporters) through the symplastic pathway. Sucrose is either cleaved or stored in the vacuole. In the cytoplasm, sucrose is either hydrolyzed again by cytoplasmic invertase into hexose or sucrose is converted into UDP-Glc by plasma membrane-related sucrose synthase and cell wall-associated sucrose synthase. However, in the cytoplasm, UDP-Glc can be converted into other polysaccharides such as cellulose, hemicellulose and callose in the presence of sucrose synthase profiles [8]. Sucrose resynthesis occurs in the cytoplasm in the presence of sucrose phosphate synthase, which then enters vacuoles for storage (see Fig. 1).

### 2.2 Source to sink relationship

The relationship between sucrose synthesis at the source (leaves) and storage in the stem has been reworded for a long time. Source-sink control of sucrose synthesis occurs when young sugarcane leaves absorb CO_2_ at the highest rates, supporting the earlier finding that end products suppress photosynthesis [11], which led to the hypothesis that sucrose metabolizing enzyme activity was the mechanism facilitating this association [12]. According to certain theories, sugarcane production, especially in lower growth phenomena, may show that after a certain amount of sucrose accumulation, sucrose represses the rate of photosynthesis [13]. Surprisingly, the wild type of sugarcane (*S. spontaneum*) has a photosynthetic rate that is 30%greater than that of hybrid *Saccharum* spp [14]. There is a positive correlation between the Carbon demand from sink tissues and the rate of photosynthesis and sucrose imported by the sink [15]. This finding suggests that photosynthesis rate depends on sink (stems) demand. When the sucrose reaches a certain level, the photosynthesis rate slows down due to a negative feedback mechanism, which means sinks may regulate sucrose accumulation in sugarcane [16]. In spite of all of this data, not much is understood about the connection between sink consumption and sugarcane photosynthetic rates. Hence, this section helps to better understand how supply and demand interact to improve sugarcane's sucrose content. In stems, sucrose accumulates in accordance with their capacity, the availability of sources and sink demand. A “source-limited plant” is one in which source availability is insufficient to satisfy sink demand. On the other hand, a sink-limited plant is one in which source availability exceeds sink demand. Most sugar plants have limited sink capacity [17]. Sugarcane stems have more potential to accumulate sucrose. Sucrose concentrations in cane stems range between 400 and 700 mM and the highest sucrose concentration is 30% of fresh weight [18]. However, on a dry matter basis, it was between 500 and 560 mg/g [19]. In young culms, 66% of sugar is consumed for respiration, protein synthesis and fibers; 34% of sugar is stored as sucrose, but the situation is reversed in mature culms [20]. Most of the Carbon in mature cane stems is split between fiber and sucrose. Sucrose production and sink capacity are governed by how sucrose is distributed across sugarcane stems. Photosynthesis contains several metabolic processes that maximize the practical use of light, Nitrogen and Carbon resources. Sink strength controls the activity of photosynthesis by a signal route that monitors the level of Carbon or Nitrogen in the entire plant and regulates the expression of photosynthesis related genes as well as the growth of leaves, rather than only through a sugar feedback mechanism [21]. At maturity, sucrose reaches a certain limit in the stem, triggering a mechanism called the negative feedback mechanism, which leads to a decline in photosynthesis rate [22]. By shading the leaves, the source-sink connection in sugarcane was investigated. The sink strength rose when all leaves were shaded except for one, which in turn upregulated source activity. Sucrose concentration in immature internodes decreased after the leaf shade treatment, which increased sink demand and feedback-upregulated source activity. Even when the leaves were enclosed, sink (stalk) sucrose accumulation was not affected [15]. It seems that the sucrose concentration in sugarcane stems is determined by sink strength rather than source limitation. During source-to-sink coordination, genes related to photosynthesis and sugar transport were controlled. The overexpression of genes, particularly those for PEPase and rubisco, may increase the activity of photosynthesis [23]. Hexokinase, an inhibited gene, may detect hexose signals to control source activity. Additionally, it showed that CO_2_ carboxylases were the primary controlled sites during source-sink coordination in sugarcane. Remarkably, the increased photosynthesis caused by the leaf shade treatment might offset the photosynthetic barrier caused by the sucrose spraying by regulating the levels and activities of PEPase and rubisco [24]. A recent study on gene association with sucrose concentration in sugarcane revealed that at maturity stage, reducing sugar declined with crop growth while sucrose enhanced. It was further stated that the elevated PEPase gene expression supported the idea that increased sink demand leads to an increased photosynthetic rate, which in turn affects the source activity [25]. Clarifying the signal molecules and genes involved in source-sink coordination in sugarcane can help boost sucrose output using the highly efficient C4 photosynthesis, but in this regard, extensive research is required.

### 2.3 Sugarcane and metabolic regulation theory

**Fig. 2:**
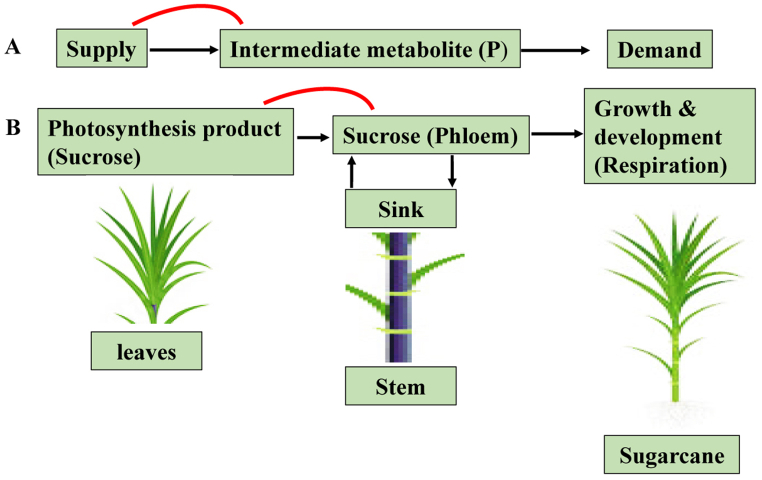
(A) Basic metabolic supply-demand system model adapted from Ref. [26]. The feedback inhibition of the supply process (red line), which is exerted through the concentration of the intermediate (P), is what regulates how much product is formed. This equilibrium between supply and demand, in turn, determines the concentration of the intermediate (P). (**B**) Demand in plants is probably the result of metabolic processes such as growth and development, respiration and storage, especially in species with high sucrose storage levels like sugarcane. As a result of the supply's sensitivity to intermediate concentrations in this example, sucrose feedback inhibition is suggested.

Ernest Munch first formulated the “pressure-flow postulates” in 1930. This theory states that the highest sucrose concentrations inside leaf cells result in the production of osmotic potential (movement of water into the phloem creates a high-pressure potential (Ψp), aka high turgor pressure, in the phloem.), which draws water into phloem cells and increases their hydrostatic pressure. These areas have lower phloem turgor as a result of sucrose being released from the phloem in stems and water flowing down the corresponding osmotic potential. A mass movement of sugars (sucrose and other sugars are transported within phloem due to a continuous flow of water and dissolved nutrients between the source and sink) is pushed from source (leaves) to sinks (stems) via interconnected phloem tubes due to the considerable variation in phloem turgor (the potential of water molecules to move from a hypotonic solution (more water, less solutes) to a hypertonic solution (less water, more solutes) across a semi permeable membrane) between the source and sink. The phloem network, which transports sucrose from leaves to stems, is a living structure rather than merely a conduit. The cells surrounding the phloem store sucrose in vacuoles to keep a consistent supply even when the source is dormant. Some of the phloem sugar is used for tissue maintenance and development. In order to identify flux and the elements that control sugar content, the behaviour of a simple, hypothetical metabolic supply and demand system was evaluated [26]. In order to prevent either a reduction in quantity or the destruction of the intermediate (sucrose), it is necessary for a closed system to control production through the supply of intermediate consumption. Feedback signalling is not fundamentally required here, though it usually is when the source is a complex metabolism. The regulation of flow through the metabolic system is governed by the concepts of supply and demand sensitivity. The supply block and the demand block's substrate combine to generate the intermediary P in their model system. Supply and demand can interact through mass action kinetics, despite the fact that this model assumes closed-loop feedback regulation by a metabolic system component (for instance, intermediate P in Fig. 2A). This paradigm can be applied to plants, with photosynthesis in leaves serving as the supply, growth and development, respiration and storage serving as the demand and sucrose in sugarcane serving as the intermediate. When it comes to sugarcane plants, it may be necessary to treat the mature stalk individually because it contains a significant amount of sugar (Fig. 2B). In response to variations in sink activity, which includes both storage and rapid usage for growth and respiration, this later model allows for alterations in the rate of transmembrane flow [27]. In turn, this might alter the leaf's sucrose pool and trigger feedback communication in the tissue that is the source of photosynthesis, controlling the process. The sugarcane stem has the greatest capacity for sucrose accumulation because there is an excess supply for development and respiration. The demand elasticity is therefore expected to be very low in the adult stem. As a result, the sugarcane plant's supply elasticity is significant and essential for the improvement of sucrose accumulation [22]. It is common for different enzymes to act as metabolic flux regulators [28] and the likelihood that any one enzyme could control the metabolic flux in a way that was significantly larger than how it affected the concentrations of related intermediates (sucrose) is high [29]. The concentration of related sugars changes more due to enzyme activity shifts in a pathway than flux, influenced by genes and proteins involved in biochemical pathways [29]. The modelling of the pathways and regulatory elements for sucrose concentration in the stem sink will be aided by these gene products [30].

### 2.4 Phloem loading strategies

**Fig. 3:**
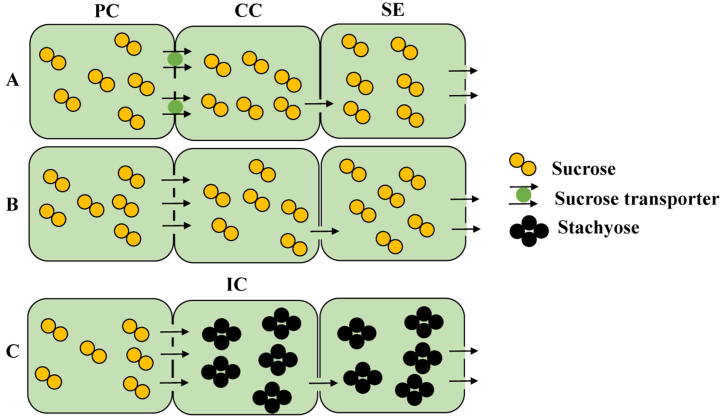
Hypothetical model of phloem loading mechanisms in plants: (A) apoplastic loading, symplastic loading, and polymer entrapment. Apoplastic loading involves sucrose entering the cytoplasm via the cell wall via the proton motive force, requiring transmembrane protein transporters. (B) Symplastic loading involves sucrose transporting cells to cells via plasmodesmata, allowing carbon to move into the phloem or companion cell complex without being transported against a concentration gradient. (C) Polymer entrapment involves an energy-intensive step where sucrose enters specialized partner cells that interact symbiotically with photosynthetic cells. Raffinose and stachyose produced from sucrose are too large to disperse back via plasmodesmata. **Abbreviations**: Photosynthetic cell (PC), companion cell (CC), intermediary cell (IC) and sieve element (SE). Arrows point out the directional flow of sugars via cells.

The processes governing the flow of sucrose from the source to the sink are not simple channels but rather a collection of sophisticated networks interconnected with other pathways [31]. There are three routes for phloem loading in plants, as follows [32]: First, apoplastic loading (sucrose enters the cytoplasm via the cell wall by proton motive force; that's why it is also called active transport) is the most significant and categorical phloem loading mechanism. Transmembrane protein transporters facilitate sucrose's entry into and exit from the apoplast (Fig. 3A). Second is symplastic loading (sucrose transports cells to cells via plasmodesmata by simple diffusion. That's why it is also called inactive transport); Carbon can move into the phloem or companion cell complex (CC) without being transported against a gradient of concentration (Fig. 3B) [33]. The third mechanism, polymer entrapment, is comparable to symplastic loading except that it includes an energy-intensive step [34]. At this point, sucrose enters specialized partner cells that interact symbiotically with photosynthetic cells, referred to as intermediate cells. Raffinose and stachyose, which are produced from sucrose, are believed to be too big to disperse back via the cell-to-cell connection known as plasmodesmata (Fig. 3c). In sugarcane, phloem loading may occur via an apoplastic or symplastic mechanism [27]. For the transportation of sucrose, it needs sucrose carriers, or transporters. In addition to apoplastic phloem loading at the leaf source, sucrose transporters have been found in many species to efflux sugar into sink parenchyma [35]. Mesophyll cells, which are photosynthetic cells in leaves, create sucrose from Carbon fixed during photosynthesis. The light responses of photosynthesis in some plant species, especially C4 plants like sugarcane, maize and sorghum, are divided between two cell types: mesophyll and bundle sheath cells [36]. In these situations, plasmodesmata, which link the cytoplasm of adjacent plant cells, allow intermediates in Carbon assimilation to flow between mesophyll and bundle sheath cells [37] Sucrose is translocated symplastically into the bundle sheath and then into the phloem parenchyma cell near the companion cell (CC)-Sieve element (SE) complex. The best example is the poplar tree, where sucrose is translocated from source to sink via symplastic routes [38]. These types of plants are called passive or inactive phloem-loading species. Because sucrose can enter the sieve tube without energy prior to long-distance transit to sink tissues. The route that sucrose follows to enter the phloem is highly variable in different plant species, but in sugarcane, sucrose follows either apoplastic or symplastic pathways in different cell subpopulations [27,39,40]. In active or apoplastic phloem-loading species, the CC-SE complex is segregated from neighboring cell types; therefore, sucrose must be discharged from the symplasm prior to entering the CC-SE complex for transportation via the sieve tubes. On the other hand, in the symplastic pathway, maximum sucrose concentrations were found in the CC-SE complex than surrounding cells, showing that it is actively loaded against its concentration gradient [41,42]. This needs energy to break down adenosine triphosphate (ATP) to generate a proton gradient across the plasma membrane of the CC-SE complex [40,43]. The proton motive force, which travels down the electrochemical potential, is utilized to move sucrose up its concentration gradient. The sucrose uptake transporters, such as sucrose carriers (SUCs) or sucrose uptake transporters (SUTs), the sugars will eventually be exported transporter (SWEET) gene family and tonoplast sugar transporters (TSTs), are responsible for this co-transport in their role as proton sucrose symporters [40]. Therefore, sucrose transporters are essential for and probably contribute to the source strength limitation on apoplastic phloem loading in sugarcane.

### 2.5 How does sugarcane temporarily store sucrose in leaf vacuoles?

More photoassimilates are produced by leaves during the day than the phloem can transport; therefore, extra photoassimilates must be temporarily stored and then mobilized to sink tissue to meet metabolic needs at night. Starch is the primary form of fixed Carbon stored in the chloroplast, which is subsequently broken down into hexoses and resynthesized into sucrose before being exported, depending on the plant species and growth conditions [44]. Sucrose can be temporarily stored in the vacuole and build up to large concentrations, necessitating movement against its concentration gradient [45]. This is achieved by distinct types of sugar transporters called tonoplast sugar transporters (TSTs), which function as sugar proton-antiporters [[46], [47], [48]]. Compared to the cytoplasm (cytosol), the vacuole's pH is much lower. Tonoplast transporters thereby link the import of sucrose from the cytosol into the vacuole with the export of a proton from the vacuole. Moreover, TSTs are crucial for the transient storage of sucrose in source leaves as well as the permanent storage of sucrose in sink tissues. How does the sucrose get out after being temporarily stored in sugarcane leaf vacuoles? In order to answer this, it is necessary to first comprehend the sucrose transporter family (SUTs). In every plant species, there is at least one member of the SUTs family localized to the tonoplast [[49], [50], [51], [52]]. In sugarcane, the role of SUTs and TSTs is sugar transportation and storage [53]. SUTs, as sucrose-proton symporters, export sucrose to the cytosol by co-transporting a proton down their electrochemical potential. The proton motive force across the tonoplast supplies the energy required for the coordinated activities of the TSTs importing sucrose into the vacuole and the SUTs sending it back out to another cell.

### 2.6 Role of sugar carriers or transporters

In higher plants, sugars are moved from leaves to stems or sink tissues with the help of different sugar transporter proteins or genes. These fundamental sugar transporters play a crucial role as sugar effluxers in the sucrose transport pathway (Chen, 2014). Sugar transporter gene families are present in different plant species, including monosaccharide transporters (MSTs), sucrose transporters (SUTs) and sucrose carriers (SUCs) [33,54]. Another family of sugar transporters is (the sugars will eventually be exported transporter) SWEET protein family, which has been reported in many important plant species. The only SUT-type sucrose transporter, *shSUT1*, has been identified in mature leaves and internode tissues of sugarcane, according to Ref. [55]. Further evidence comes from the discovery of six sucrose transporter members using artificial bacterial chromosomes, which led to the projection that *SUT1* and *SUT4* would be the primary sucrose transporter family members. These genes exhibited extensive expression in sugarcane under a range of conditions [56]. Sucrose transport from source to stem depends on sugar transporters that facilitate the transport of sucrose, which is correlated with the allocation of sucrose in the storage tissue. Another study on sugarcane demonstrated that the *ShSUT1* function recovers sucrose from intercellular spaces for transport and storage but is not directly involved in phloem loading [57]. [58] revealed five sucrose transporter genes (*SoSUT1–SoSUT5*), which were transcribed during the sucrose accumulation process in sugarcane culm. In sugarcane, 22 of SWEET's member proteins were identified [59]. Among the 22 genes, *SWEET1a/4a/4b* and *SWEET13c* contribute higher accumulations of sucrose in stalk and leaf (mature and immature); however, *SWEET13c* was found to be a major contributor to the efflux in the transportation of immature, ripening and mature stalks, suggesting their tissue-specific role from source to sink and being considered a promising option for sucrose transportation in tissues of sugarcane plants, starting at the source and ending at the sink. Recently, 105 sugar transporter genes were revealed in two sugarcane species, *S.spontaneum* and *S.officinarum* [60]. Numerous genes involved in the source-to-sink process have been found, revealing some of the possible causes of sugarcane's high sucrose accumulations [61]. Collectively, sucrose transporter genes during sucrose transportation and storage play a vital role in plants. Particularly, the *SWEET13c* gene may help transport sucrose from source to sink, resulting in greater sucrose accumulation in sugarcane stems. When sucrose is synthesized, the sucrose is temporarily stored in the leaf vacuole. Then the second step is sucrose entering the phloem cell. To transport a long-distance pathway from leaf to stem, many ways are possible such as apoplastic or symplastic. When sucrose is synthesized, the sucrose is temporarily stored in the leaf vacuole. Then the second step is sucrose entering the phloem cell. To transport a long-distance pathway from leaf to stem, many ways are possible, such as apoplastic or symplastic [62]. Finally, sucrose is unloaded into the apoplasmic or symplastic and then taken up into the sink cell stem. This mechanism is not simple; there are many genes or proteins that can play an important role during this process. Sugar transporters play a role in a number of physiological and metabolic processes in other plant species, as presented in Table 1. These findings suggest that tonoplast transporters are very important for the higher accumulation of sucrose in the stem vacuole. In addition, these findings provide a theoretical basis for sugar carrier or transporter research and enrich potential gene resources for genetic improvement and molecular breeding of sugarcane. The study utilized expression analysis to identify high-expression sweet genes in plants, hypothesizing their potential contribution to sucrose transport.

**Table 1:** Functions of sucrose transporters genes exist in different plant species. This table denotes the sucrose transporter genes and their functions in various plant species.

Organism Name	Gene	Functions	References
***Zea mays***	*SWEET13a, b, c*	Effluxing sucrose to the apoplasm	[63]
	*ZmSUT1*	Apoplastic loading	[35]
***Gossypium hirsutum***	*GhSWEET*	Cell expansion and development Response to biotic and abiotic stresses	[64]
***Arabidopsis thaliana, Brasica rapa and Nicotiana attenuata***	*SWEET9*	Nectar secretion and transporter	[54]
***Arabidopsis thaliana***	*AtSWEET13-14*	Modulating gibberellin (GA) response	[65]
	*AtSWEET11,12,13*	Sucrose efflexers	[40]
	*AtSUT1*	Sucrose exporter	[35]
	*AtSUC4*	Sucrose export from the vacuole	[66]
	*TMT1/2*	Hexose loading into vacuole (antiporter)	
***Oryza sativa***	*TSTs*	Sucrose accumulation in vacuoles	[67]
	*OsSWEET11*	Grain filling and sucrose transporter	[68]
	*OsSUT1*	Sucrose transporter	[69]
***Sweet sorghum***	*TSTs and SWEETs*	Sucrose accumulation and transporter	[70]

### 2.7 Sucrose post transcriptional regulation in sugarcane

The mechanism known as SIRT (Sucrose-Induced Repression of Translation) is an important and common phenomenon in higher plants [71]. Many plant species indicate the existence of transcriptional and post-transcriptional regulators, especially the *S bzip* gene family [33] This *S bzip* gene contains uORF in its mRNA; this gene is regulated by sucrose signaling [72].Understanding the function of the regulated transporter-like protein (ZIP) gene family, which controls target genes' *bzip11,bzip44* and *bzip53* transcription, is crucial for understanding source-sink coordination regulation [33].Sugar serves as a signaling molecule that regulates the transcription of many plant genes by maintaining the stability of mRNA and proteins. Sucrose inhibits the main open reading frame (ORF) of *Atbzip11*, which encodes a transcription factor from the small family known as a basic Leucine zipper [73]. The post-transcriptional regulatory mechanism is known as “sucrose-induced repression of translation (SIRT)". This SIRT is made possible by one of the upstream open reading frames (uORFs) found in the unusually long 5′ leader region of the *S bzip* gene, like the *Atbzip11* transcript [74]. There is a significant degree of similarity between these uORFs and those identified in the *Atbzip1, Atbzip2, Atbzip44,* and *Atbzip53* genes, which are all members of the Arabidopsis small *bzip* family [75]. The sucrose regulated upstream on the reading frame is the name given to it, identifying it as belonging to the bzip gene family's lip19 subgroup. Thus, the term “sucrose controlled upstream on the upstream open reading frame” is used to identify the lip19 subgroup of the *S bzip* gene as the home of numerous plant species' S *bzip* homologues. The *S bzip* gene supports the SIRT process by maintaining a conserved SC-uORF [71]. When the endogenous sucrose levels reach their threshold level, initiating SIRT due to the negative feedback mechanism. Therefore, this finding proposes that sucrose is a signaling molecule that regulates the translation of the S *bzip* gene's main open rereading frame (ORF). In this post-transcriptional system, it is probable that SIRT helps maintain the homeostasis of sucrose and that the SC-uORF serves as a sucrose sensor (via a negative feedback mechanism). A similar post-transcriptional mechanism was described in Arabidopsis [76]. Additionally, *KIN10* and *KIN11* are kinase proteins that serve as important signal integrators for adjusting to low energy, including darkness, low sugar and stress conditions [77]. There is strong proof that a leucine-rich repeat (LRR) receptor-like kinase is involved in sucrose accumulation in different cane varieties, which suggests its contribution to controlling sucrose accumulation [78]. Particularly, S *bzip* is responsible for facilitating the *KIN10* kinase-signal pathway and activating the transcription of Asparagine Synthase (*ASN*). According to Ref. [79], Asparagine Synthase (*ASN*) transcription activation is stopped by sugars (sucrose and glucose); the reason is that those sugars downregulate the *KIN10-11* protein activation [80]. Furthermore, overexpression of *tbz17* was positively associated with sucrose phosphatase synthase and *FBPase* genes [71]. Recently [81], reported the *bzip* family gene *Scbzip44* in *S. spontaneum*, which is closely related to Arabidopsis *Atbzip44*, which responds to sucrose sensing during sucrose accumulation in sugarcane. *S bzip* genes are present as evolutionarily conserved transcriptional factors in eukaryotes. This S1-group *bzip* gene forms heterodimers with C-group members to form a signaling hub in the model plant (Arabidopsis) [82]. These allow for metabolic adaptability and survival in response to stress by facilitating metabolic reprogramming during an energy shortage [83]. The five S1-group *bzip* genes that are encoded by Arabidopsis are *Atbzip1, Atbzip 2, Atbzip 11, Atbzip 44,* and *Atbzip 53*. They all have a highly conserved uORF in their mRNA's 5′ UTR [74]. The translation of these genes (*S bzip*) is repressed by sucrose (when sucrose reaches a certain level in stems in the case of sugarcane, then due to negative feedback inhibition, the SIRT inhibits sucrose accumulation), resulting in the phenomenon referred to as sucrose-induced suppression of translation (SIRT), and this inhibition depends on the conserved upstream peptide sequence [84]. Additionally, a functioning SIRT mechanism has been shown in gymnosperms, and these peptides are preserved in a wide range of plants [85]. This idea was implemented on the *S bzip* transcription factor of strawberry fruit to enhance the sucrose content [86]. It is noteworthy to mention that a transcriptome investigation conducted on sugarcane (*Saccharum officinarum*) has demonstrated a favorable correlation between the expression of genes encoding the sucrose transporter, ornithine aminotransferase (OAT), and ASN and the sucrose concentration. Both genes ASN and OAT are associated with proline metabolism, dehydrin metabolism, and sucrose metabolism. This phenomenon was found in the high sucrose content of stems. *S bzip* genes such as *Atbzip11, Atbzip53, Tbz17,* and *Scbzip44* transactivate the ASN and KIN10 and induce metabolic reprograming, which turns into activation of the sucrose synthase pathways (SPS and SPP) [71,80].

**Fig. 4:**
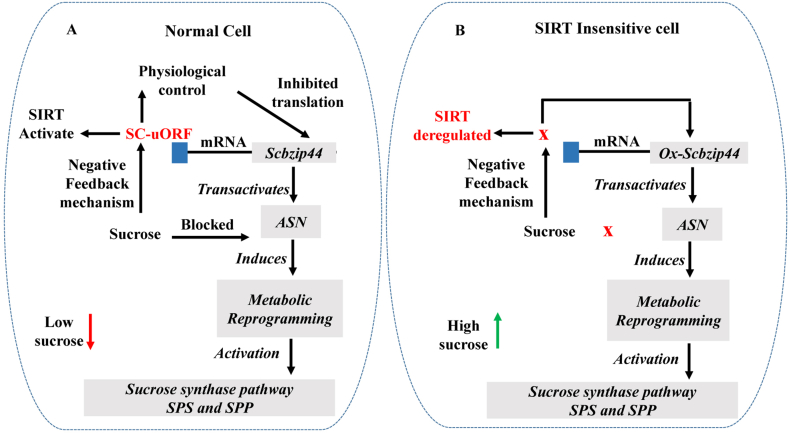
A hypothetical model of the mechanism known as sucrose-induced repression of translation (SIRT) is intended to elucidate how expression of the “*Scbzip44″* gene in sugarcane plant cells accumulates maximum sucrose (A) In normal cells, the *Scbzip44* gene works as a transactivator for the ASN gene, and the ASN gene activates the metabolic reprogramming pathways. However, after a certain level of sucrose accumulates in sugarcane stems due to the negative feedback inhibition signaled by sucrose to Sc-uORF, which inhibits the *Scbzip44* gene translation, sucrose is limited in this cell. (B) On the other hand, in SIRT-insensitive or deregulated cells with overexpression of the *ox-Scbzip44* gene, sucrose accumulates continuously without disruption, so maximum sucrose is obtained. **Abbreviations**: Asparagine synthase (*ASN*), Sucrose phosphate phosphatase (SPP), Sucrose phosphate synthase (SPS), Sucrose-controlled upstream open reading frame (SC-uORF), Overexpression of sugarcane zip transcription factor gene (*Ox-Scbzip44*).

In light of the available data, it is suggested that a hypothetical model for explaining sucrose accumulation in *S bzip* family genes like *Scbzip44* in sugarcane plants be developed. In sugarcane plant cells, *Scbzip44 transactivates ASN* and induces metabolic reprogramming. This causes the “sucrose synthesis pathway” to become active. If the sucrose concentration reaches its upper threshold level, SIRT is activated and inhibits the translation of *Scbzip44* through a negative feedback regulation mechanism. The amount of sucrose that accumulates in sugarcane stems is limited by this negative feedback mechanism. Contrarily, in the SIRT-inhibited *Scbzip44* sugarcane plant, even when sucrose concentration reaches its maximum level, *Scbzip44* translation continues because SIRT is not activated. *A*ccording to a hypothetical scenario in which normal sugarcane cells express the endogenous *Scbzip44* gene and its transcribed product contains SC-uORF (at the 5′ leader region on mRNA), induced SIRT keeps cellular sucrose concentration at a limited level when the SC-uORF of the *Scbzip44* transcript senses sucrose (Fig. 4A). In the later cell, the *Ox-Scbzip44-insensitive* gene (without SC-uORF) transactivates the *ASN* gene and induces metabolic reprogramming, which activates the sucrose synthase pathways and leads to maximum sucrose accumulation (Fig. 4B).In a transcriptional-based study on sucrose content, around 34,476 genes were observed to be deferentially expressed in high and low accumulation genotype stems (mature and immature), suggesting that the majority of gene expression is consistently connected with sucrose accumulation [87]. These findings propose that sucrose may have a signaling role in sugarcane growth and development in addition to being a main metabolite and a storage and transport sugar. Recently, a transcriptome study revealed that 18,722 genes were differentially expressed during sucrose accumulation in two different genotypes of sugarcane. Most differentially expressed genes (mRNAs) were involved in starch, sucrose metabolism and photosynthesis [88]. Another study hypothesized that microRNA may contribute to sucrose accumulation in sugarcane, but not by directly targeting the genes involved in sucrose metabolism but rather by specifically regulating transcription factors from the formative to the maturity stages [89]. These findings suggested that microRNAs indirectly regulate transcriptional factors (such as *Scbzip44* in sugarcane), which may play a very important role via metabolic reprogramming in sucrose accumulation. It is suggested that making SIRT insensitive through overexpression of a *S bzip* family gene such as *Scbzip44* is a novel strategy to generate transgenic sugarcane crops that may increase endogenous sucrose levels. This study provides us with a better understanding of the sugar regulatory mechanism and its content in sugarcane.

#### 2.7.1 Function of sucrose metabolizing enzymes

Sucrose-metabolizing enzymes play a vital role during the processes for sucrose production, transportation and storage. Sucrose metabolizing enzymes in sugarcane plants are categorized into two main groups: the sucrose synthesis group, which includes sucrose phosphate synthase (SPS), and sucrose phosphate phosphatase (SPP). On the other hand, the sucrose hydrolyse group includes cell wall, cytoplasmic, and vacuolar invertase, each of which exists at different locations with a different pH [90]. However, sucrose synthase (SuS) catalyzes the reversible hydrolysis of sucrose into hexose sugars or the resynthesis of sucrose and other polysaccharides. During the sucrose regulatory mechanism, at various growth phases, sugarcane depends on these enzymes. Hereinafter, we try to review the important characterizations and functions of each enzyme in sucrose metabolism.

#### 2.7.2 Sucrose phosphate synthase (EC 2.4.1.14)

Sucrose Phosphate Synthase (SPS) is a key rate-limiting enzyme in sucrose synthesis, catalyzing the transfer of fructose-6-phosphate (F6P) from the acceptor to the donor, uridine-diphosphate glucose (UDPG). This enzyme is essential for the metabolism of sucrose [91]. SPS is regulated by a complex mechanism consisting of allosteric regulation and phosphorylation regulation [92]. Additionally, the activator and inhibitor of the regulation are, respectively, glucose-6-phosphate and inorganic phosphate. The amount of glucose-6-phosphate, the activator, also has a role [93]. SPS is encoded by the SPS gene family and activity is controlled at the transcriptional as well as the enzymatic level via allosteric activation [94]. When sucrose is synthesized by SPS, parenchyma cells in mature sugarcane stems have a maximal capacity to retain sucrose. The highest concentration of sucrose in the storage stem is generally assumed to be the sum of the actions of sucrose transport, sucrose phosphate synthase production, and invertase-catalyzed sucrose hydrolysis. Several studies demonstrate a positive relationship between sugarcane cultivar growth profiles and sucrose content or concentration rate [8]. When compared to low sucrose cultivars and immature internodes, high sucrose content cultivars and mature internodes have higher sucrose phosphate synthase activity [95]. Previous studies stated that two types of isoforms, *SoSPS1 and SoSPS2,* are found in the leaves, while *SoSPS2* is found in all tissues in sugarcane plants [96]. In transgenic sugarcane, upregulation of the sucrose phosphate synthase gene (*SoSPS*) increased the sucrose concentration, increased plant height and increased the number of tillers [97]. It was also noted in rice plants with overexpressed *SoSPS1* [98]. Conversely, downregulating SPS expression in Cucumis melo causes a decrease in sucrose content of over 7% as well as the similar effect on biomass production [99]. Different kinetic forms of sucrose phosphate synthase are present in sugarcane internodes at different phenological stages [8]. Currently, in sugarcane, the Sucrose phosphate synthase-B (SPSB) genes or alleles (39 halophytes) have been linked to sucrose accumulation. The sucrose phosphate synthase gene (*SPSB6*) is considered the best sucrose accumulation haplotype among all the thirty-nine haplotypes [100]. This data indicated that not only a single SPS gene is involved in sucrose synthesis, but multiple alleles also contribute to sucrose synthesis. Further, the activities of the five sucrose synthase genes, namely *SPS1, SPS2, SPS3, SPS4,* and *SPS5*, were higher in sugarcane (juvenile or immature) internode tissues. But there is no proof of enhanced transcription in the *SPS5* isoform in the ripening stage [101]. This finding proposes that factors other than transcription determine the SPS's enzymatic activity. For example, during sucrose metabolism, there is a single-point mutation showed at the UDP-Glc binding site that causes the enzyme's activity to decrease. This finding implies that the catalytic activity of SPS in sugarcane plants is influenced by these residues. Additionally, comprehension of the UDP-Glc bidding site offers fresh perspectives on enzyme engineering and redesigning catalytic processes for UDP in sucrose-regulating mechanisms [102]. In sugarcane, sucrose concentrations naturally exhibit declining invertase expression and maximum SPS expression rates, along with high sucrose contents [103,104]. However, low sucrose content varieties have found maximum invertase activity. In contrast, maximum SPS activities are exhibited in high sucrose content varieties. Hence, SPS plays a vital role in sucrose accumulation, growth, and development through sucrose metabolism. It is not only present in sugarcane but also in other plant species as well. Recently isolated SPS isoforms are presented in Table 2.

**Table 2:** Sucrose phosphate synthase isoforms exist in different plant species.

Organism Name	Gene Isoforms	Functions	References
***Zea mays***	*ZmSPSΔ482*	Enhance source capacity	[107]
***Malus domestica***	*MdSPSA2.3*	Sucrose accumulation	[108]
***Castanea***	*SPS*	Sugar metabolism	
***Actinidia chinensis and A. eriantha***	*AcSPS1-AcSPS2 AcSPS4-AcSPS5*	sucrose accumulation during fruit development stage	[109]
***Manihot esculenta Crantz***	*MeSPS1–MeSPS5*	Starch accumulation in the root	[110]
***S. spontaneum and S. saccharum***	*SsSPSA-SsSPSC, SsSPSD1-SsSPSD2*	Carbohydrate metabolism and sucrose accumulation	[111]
***Sorghum bicolor***	***SoSPSA-SoSPSC, SoSPSD1-SoSPSD2***	Carbohydrate metabolism and sucrose accumulation	
***Ananas comosus***	*AcSPS*	Sucrose synthesis	[112]

Sucrose phosphate synthase expression is affected not only by transcriptional and enzymatic levels but also by other abiotic factors like temperature. Enzyme is sensitive to temperature fluctuations, which affect the enzymatic activities in plants; optimum temperature is very important for the sucrose-accumulating stage in the sugarcane stem. The temperature below 8 °C changes the enzymatic activities, either synthesizing or hydrolyzing, at the ripening stage [105]. The optimal daily temperature of 12–14 °C is best for higher sucrose accumulation. The most optimal temperature range is 30–32 °C for sugarcane growth and development. But more than 34 °C declines the plant's growth; at 0 °C or below, sugarcane suffers a chilling injury [106]. However, a study on the stress physiology of sugarcane under heat stress (at 45 °C) conditions reveals that sucrose concentration in sugarcane cultivars is adversely impacted. This result suggested that when temperatures are exceeded, reactive oxygen species increase in plant cells, enzymes denature and they cannot continue their function, resulting in a low sucrose yield. The evidence presented above suggests that sucrose phosphate synthase is essential for sucrose metabolism in plants, particularly sugarcane. So, a deep understanding of the role of this enzyme is needed for sucrose accumulation in sugarcane.

#### 2.7.3 Sucrose synthase (EC 2.4.1.13)

Over the past three decades, extensive research has led to new insights into the control and role of sucrose synthase in the metabolism of sucrose. The enzyme sucrose synthase (SuS), whose protein's structure are frequently thought of as homotetramers [113], is a member of the glycosyltransferase subfamily. In the presence of NDP-glucose, it facilitates the reversible hydrolysis of sucrose into fructose [114].

(NDP-glucose) + d-fructose ⇌ NDP + sucrose

This enzyme's substrates are NDP-glucose and d-fructose, while its products are d-fructose and sucrose. For the synthesis of starch, primary metabolites and energy, sucrose cleavages by sucrose synthase enzymes produce a variety of compounds. In the cytoplasm, where SuS plays a significant role in sucrose metabolism, they hydrolyse sucrose into UDP-Glc, cellulose, hemicellulose and other polymers and hexose by invertase in sugarcane [115]. Numerous organelles, including the vacuole membrane in red beet, the golgibody in poplar and maize, the mitochondria in corn and the plastid in Arabidopsis seed, expressed sucrose synthase [116]. Sucrose synthase (SuS) isoforms have been identified in the cell walls of several plant species, including rice, which contains six [117] and grapes and sugarcane, which each have five of these described gene isoforms [118]. As a result of recent advances in plant genome sequencing and assembly, as well as the publication of more draft genomes, the sucrose synthase gene family has been characterized in a wider range of plant species and in greater depth. The isoform functions of the other plants, along with sugarcane, are presented in Table 3.

**Table 3:** Sucrose synthase isoforms exist in different plant species.

Organism Name	Gene Isoforms	Functions	References
***Sugarcane***	*ScSusy1 - ScSusy5*	Sucrose catabolism	[129]
***chestnut nut***	*Eleven SuS*	Sugar metabolism	[108]
***Sorghum Bicolor***	*SbSusy*	Sucrose metabolism	[130]
***Banana- Fenjiao***	*MbSUS-2.1 - 2.4*	Starch accumulation	[131]
***Banana-Baxijiao***	*MaSUS-2.1-2.2, 2.3 MaSUS-3.2*	Starch accumulation	
***Chinese-pear***	*Thirty SuS*	Fruit development and resistance to stress	[132]
***Solanum lycopersicum***	*SlSUS1- SlSUS4*	Regulation of plant growth and involved in Auxins signaling pathway	[116
***Vitis vinifera* L**	*VvSS1-VvSS5*	Resistance to biotic and abiotic stresses	[118]
***Gossypium***	*GaSus1- GaSus7*	Fiber growth and development	[133]
***Arabidopsis***	*SuS1-SuS6*	Cellulose and starch synthesis	[134]

The main function of this enzyme is to promote plant growth and development. However, different abiotic stresses severely affect the enzymatic activity and sucrose contents. Previous studies explained that upregulation of sucrose synthase in potato plants caused increased growth and development that increased starch contents [91]. This study suggests and confirms its significance in sucrose metabolism and sink strength as a potential candidate gene for the development of agronomic characteristics in plants [119]. Recently, one study explained that high temperatures affect the SuS activity, which declines the sucrose content in different sugarcane varieties [105]. A limited tolerance for stressful situations is said to hinder growth and development when SuS activity is restricted. Drought stress reduced SuS activity, resulting in lower grain yields in Maize [120] and sweet potato [121]. In carrots, sucrose synthase (DcSus) activities were continuing to decline when the sucrose content observed an increasing trend. This indicated that DcSus activity was directly associated with sucrose accumulation [122]. So, this study will be helpful in providing effective insights for sucrose accumulation in sugarcane plants. The activity of the SuS enzyme is controlled by two phosphorylation sites, including a serine site at positions 11–15 that is assumed to be necessary for membrane attachment and another serine site at around position 170 that is expected to influence protein breakdown. This protein phosphorylation (Rsus-3) may increase the activity of rice sucrose synthase [123]. It was revealed that two glutamate residues (E678 and E686) and a phenylalanine residue (E680) are necessary for enzyme activity in the RSuS3 protein [124]. But the SuS tetramer isoform (*SUSS1*) is found in Arabidopsis plants [125]. It's regulated by pH, with sucrose synthesis occurring at pH 7.5–9.5 and cleavage occurring at pH 5.5–7.5 [113] and it also maintains high temperatures in plants [126] There is no increase in sugar concentration due to the regulation of sucrose synthase activity, but there is apparent growth retardation. However, it was concluded that the potentials must still be explored by modifying the embryonic behaviour of a specific sucrose synthase and its isoform in plant species adapted for maximum sucrose concentration [127]. Sucrose concentration and sucrose synthase activity are positively correlated in sugarcane developmental profiles [128]. In the stem of the sugarcane plant, there are three distinct sucrose synthase isoforms, each with unique kinetic characteristics and expression patterns. The ratio of sucrose synthesis to hydrolyse activity increases by 41% as sucrose accumulation increases in the stalk, whereas fructose concentration decreases by 7.5%. The sucrose flow and concentration are determined by both enzymes and their isoform fractions. According to their control coefficients on sucrose synthesis and hydrolysis, the SuS isozymes have a negative influence on each other and on fructose phosphorylation [128]. If considerable volumes of biochemical data are available, it may be possible to develop kinetic models that estimate the optimal enzyme activity patterns for increased sucrose concentration. This finding revealed that various isoforms of SuS could have significant differential effects on metabolite content (sucrose), hence altering metabolic regulation and laying the groundwork for further research into the regulatory mechanism of sucrose content in sugarcane plants.

#### 2.7.4 Sucrose phosphate phosphatase (EC 3.1.3.24)

Sucrose phosphate phosphatase (SPP) is another name for sucrose-6-phosphate phosphohydrolase (S6PP). SPS and S6PP work together to effectively produce sucrose. The process ends with the irreversible cleavage of sucrose-6F-phosphate, which is produced by sucrose-phosphate synthase, into sucrose [135]. Two sucrose phosphate phosphatase (SPP) isoforms (*S6PP-1* and *S6PP-2D*) were more active in immature sugarcane plants than mature ones [136]. [10] reported that at the early development stages, when plant growth is quicker, there is a greater need for sugars. This result indicates that during the sucrose accumulation stage, these enzymes may play an important role in enhancing the maximum sucrose concentration in sink tissues. In tobacco plants, the strategy applied for SPP gene inhibition was RNAi, which drastically reduced sucrose levels in transgenic plants [137]. Four isoforms were revealed in the Arabidopsis plant; among the four isoforms, *SPP2* is the most active isoform, indicating higher expression, while *SPP1* is a non-active isoform. Sucrose is synthesized by the consecutive actions of SPS and SPP. So *SPP1* is a non-active isoform; this finding suggests that it may play another role or be associated with SPS or other enzymes [138]. The SPP had higher enzyme activity than the SPS; this result suggested that the SPP plays a substantial role in sucrose accumulation [139]. Additionally, the link between these two enzymes has provided more evidence that SPP may play a more important role in sucrose metabolism [140]. This finding contributes to the information regarding sucrose metabolism in sugarcane plants as well as other plants. Recently, many SPP isoform functions have been characterized in many plant species, as presented in Table 4.

**Table 4:** Sucrose Phosphate Phosphatase isoforms exist in different plant species.

Organism Name	Gene Isoforms	Functions	References
***S. spontaneum and S. officinarum***	*S6PP-1 and S6PP-2D*	Sucrose metabolism	[136]
***Arabidopsis thaliana***	*AtSPP1, AtSPP2, AtSPP3a and AtSPP3b*	Direction of sucrose synthesis	[138]
***Oryza sativa***	*OsSPP1 to OsSPP4*	Sucrose biosynthesis	[141]
***Triticum aestivum***	*TaSPP1 to TaSPP3*		
***Zea mays***	*ZmSPP1 and ZmSPP2*		

#### 2.7.5 Invertase isozymes (EC 3.2.1.26)

On the basis of subcellular localization, invertase is classified into three types: vacuolar, cytoplasmic, and cell wall [142]. On the other hand, on the basis of pH, invertase is classified into two types: cidic invertase and neutral invertase, which is also called alkaline or cytoplasmic invertase, and acidic invertase [143]. Invertase enzyme activities vary depending on temperature and pH, which range from 40 to 60 °C and pH 3–9 [144,145]. Cytoplasmic is an alkaline type, active at pH 7.5, whereas vacuolar (pH 5) and cell wall invertases (pH 3.5) are acidic in nature and have a maximal activity at 50–55 °C [146]. Invertase and its isoforms appear to be sucrose-specific in plants [147]. The biological roles of invertase isozymes are determined by the kind of tissue and its location [148]. The function of vacuolar invertase is to mobilize sucrose, regulate sugar content in the vacuole and contribute significantly to the development and proliferation of the sink tissue. Rapid growth of immature cells has a high fructose and glucose content but a low sucrose content, indicating a fundamental role in the metabolism of sucrose [149]. Cell wall invertase's function is to unload sucrose and balance the amount of sugar in tissues that serve as sources and sinks [150] by supplying the sucrose to the stem or sink through the apoplastic pathway. Another major invertase enzyme that regulates hexose sugar levels across intracellular tissues is cytoplasmic invertase, which is most likely found in the cytosol [151]. Invertases play a major role in sugarcane's regulation of sucrose concentration as well as the early phases of growth and development [152].

Invertases play a crucial role in sucrose metabolism in sugarcane crops by hydrolyzing sucrose into hexoses [153], but their activities depend on the growth stages. This could be due to the regulatory feedback mechanism between the leaf and the stem during sucrose accumulation [22]. In the vegetative and grand growth stages, invertase activities are higher than at maturity because immature plants need lots of energy for growth and development [154]. Acid invertase was high in the cell wall and vacuole of immature issues, actively growing and absent at the maturity stage [155]. In comparison to other sucrose-metabolizing enzymes, soluble acid invertase is expected to play a more important role in limiting sucrose accumulation in sugarcane internodes [156]. At maturity stages, vacuolar invertase activity declined, but both cell wall and cytoplasmic invertases cleaved sucrose together [157]. Minimum alkaline invertase levels existed in immature tissues, but maximum levels in older tissues were observed [158]. This finding suggested that cytoplasmic invertase governs the transport of sucrose from vascular to sink tissues, which increases glucose and fructose in the mature stem [159]. Soluble acid invertase activity decreased at 12 months in sugarcane, but after 12 months, the SAI activity exhibited a minor increase in internodes. Additionally, because photosynthesis is reduced after ripening, this rise may cause a drop in the content of sucrose in mature tissues by remobilizing stored sucrose for cellular growth and maintenance [160]. Invertase isozymes negatively affect sucrose concentration in various sugarcane varieties, and minimum acid invertase activity increases sucrose content during ripening. Growth-specific activities fluctuate from formative to mature, suggesting maximum sucrose accumulation may occur at maturity if invertase activities inhibit or downregulate. Many plant species have various invertase isoforms and their functions; **see** Table 5**.**

**Table 5:** Invertase and its isoforms exist in different plant species.

Organism Name	Invertase Isoforms	Functions	References
***Camellia sinensis***	*CsInvInhs*	Cold stress response Vegetative and reproductive process at post-translation level.	[161]
***Malus domestica***	*MdINVs*	Fruit development and cold stress response	[162]
***Nicotiana tabacum***	*NtINV*	Leaf growth and development Abiotic stresses (drought and salinity)	[86]
	*NtNINV10*	Sugar metabolism	
***Sorghum bicolor***	*SbSAI-2*	Sucrose metabolism	[163]
	*SAI-1*		[164]
***Fragaria X Ananassa***	*FvVIN2*	Sugar metabolism	[165]
***Ananas comosus***	*AcNI*	Sucrose hydrolysis	[112]
***Zea mays***	*INVCW7*	Starch accumulation development of the female reproductive system	[166]
***Capsicum***	*CaCWINV2 CaVINV1*	Responsible for sucrose cleavage in reproductive organs	[167]
***Populus***	*PtVINV1/2*	Responsible to salt stress	[168]
	*PtVINV1-3*	Cold stress	
***Cassava***	*MeCWINV1-3*	Sucrose catabolism	[169]
	*MeCWINVs*	Sucrose exploration from source to sink	
***Triticum aestivum***	*CWI21– CWI22*	Associate with wheat kernel weight	[170]
	*Ta-A-Inv*	Cleavage of cytosolic sucrose during cold stress	[171]

## 3 Concluding remarks

Sugarcane stem accumulates sucrose as a result of a system that combines sucrose metabolism, transportation and partitioning. There have been discoveries of sugar transporters, sucrose metabolism enzymes and potential regulator genes. However, it is unknown how the regulatory networks of these genes impact the amount of sucrose accumulation. Analysis of the interactions between the regulatory mechanisms for source-sink communication, the enzyme activities involved in the metabolism of sucrose and the role of post-transcriptional control of sucrose-induced regulation of translation (SIRT) still has to be done. Additionally, more functional research on sugar transporters and regulator genes is required in relation to sucrose accumulation in sugarcane. Sugarcane has a complex genome (due to polyploidy), making it challenging to produce mutants for complementary genetic research. For that reason, it can explore the function of the genes by altering gene expression in sugarcane genotypes with high or low sucrose content. Sugarcane has the potential to accumulate the most sucrose in its stems. Sugarcane must improve sink strength to boost sucrose productivity since sucrose accumulation is sink-limited. Understanding the sucrose molecular regulatory networks can enable us to change sink capacity via molecular sugarcane breeding. This review's highlighted work intends to close several knowledge gaps about sucrose buildup in sugarcane. Specifically, focused on the role of post-transcriptional control of sucrose-induced regulation of translation (SIRT), the function of sucrose metabolizing enzymes, the characterization of zip genes and sucrose transporter gene regulatory networks that regulate sucrose partitioning and some of the unresolved research gaps that need to be filled by future studies. According to the current review, the genes responsible for sucrose accumulation in plants are sucrose phosphate synthase, sucrose phosphate phosphatase and *S bzip* family genes, including *Scbzip44, Atbzip44, Atbzip53, Atbzip11* and *tbzip17*. While sucrose carriers and SWEET family genes are responsible for sucrose transportation. A future study is also required to identify the genes that control sucrose flow in multiple pathways, including apoplastic, symplastic and polymer trapping mechanisms from the source (leaves), as well as the genes that regulate long-distance coordination between the tissues of the source and the tissues of the sink for sucrose accumulation in can stems. There have been a number of “surprises” from the regulation mechanisms of plant sucrose pathways. For instance, it now seems certain that numerous sucrose metabolizing enzymes and probably several pathways would be active simultaneously in order to achieve maximum sucrose accumulation in a highly selected crop. To choose potential targets, however, the most knowledge possible about sucrose regulation systems is required. The sugarcane genome is extremely complex, which may disappoint expectations for a high sucrose yield, but we believe that results from the past several years suggest the potential for significant practical advantage through a connection between molecular mechanisms research, genetic engineering and biotechnological approaches in sugarcane crop sucrose regulatory mechanisms. It is suggested that making SIRT insensitive through overexpression of a *S bzip* family gene such as *Scbzip44* is a novel strategy to generate transgenic sugarcane crops that may increase endogenous sucrose levels. This study provides us with a better understanding of the sugar regulatory mechanism and its content in sugarcane.

## Additional information

No additional information is available for this paper.

## Funding statement

This work was funded by the Yunnan Seed laboratory (202205AR070001-09), the Key Research and Development Plan of Yunnan Province, a special Project of International Science and Technology Cooperation (202203AM140030),the Yunnan Science and Technology Talent and Platform Program (202205AM070001) and the key research plan of Yunnan Province ((202203AK140029).

## Data availability statement

Data included in article/supplementary material/referenced in article.

## CRediT authorship contribution statement

**Faisal Mehdi:** Writing – review & editing, Writing – original draft, Conceptualization. **Saddia Galani:** Writing – review & editing, Visualization, Formal analysis. **Kamal Priyananda Wickramasinghe:** Writing – review & editing, Data curation. **Peifang Zhao:** Writing – review & editing. **Xin Lu:** Writing – review & editing. **Xiuqin Lin:** Writing – original draft. **Chaohua Xu:** Writing – review & editing. **Hongbo Liu:** Writing – review & editing. **Xujuan Li:** Writing – review & editing, Writing – original draft, Conceptualization. **Xinlong Liu:** Writing – review & editing, Supervision, Resources, Methodology, Investigation, Funding acquisition.

## Declaration of competing interest

The authors declare that they have no known competing financial interests or personal relationships that could have appeared to influence the work reported in this paper.
